# Use of Right Ventricular Support with a Centrifugal Pump in Post-Valve Surgery Right Ventricular Failure: A Case Series

**Published:** 2014-01-12

**Authors:** Abdol Rasoul Moulodi, Gholam Reza Sheibat Zadeh, Feridoun Sabzi

**Affiliations:** Imam Ali Cardiovascular Center, Kermanshah University of Medical Sciences, Kermanshah, Iran.

**Keywords:** Cardiac surgical procedures • Ventricular dysfunction, Left • Heart-assist devices

## Abstract

The optimal treatment method for right ventricular failure after valve surgery complicated by a low cardiac output has not been determined, although several case reports have been published on patients with ventricular failure and arrhythmia who were bridged to cardiac transplantation using biventricular or left ventricular assist devices. This case series illustrates successful circulatory support of 4 patients with prolonged low cardiac outputs and right ventricular failure and arrhythmias after valvular heart surgery with or without severe pulmonary hypertension. In-hospital death occurred in one patient and 3 patients were discharged from the hospital with good general condition. At two years' follow-up, 2 patients were in functional class one but another patient underwent laparotomy for multiple splenic abscesses and died from multiple organ failure.

## Introduction

Post-cardiotomy right ventricular (RV) failure can be caused by prolonged aortic cross-clamp time and cardioplegic arrest, inadequate myocardial protection, and right coronary artery obstruction due to coronary vasospasm, air embolization, and thrombus. In transplantation recipients, donor organ ischemia and preexistent or perioperative pulmonary hypertension mainly contribute to the development of acute refractory RV failure.^1^^, ^^2^

RV failure is one of the serious complications of open heart surgery, and if it does not respond to maximum inotropic agents and the intra-aortic balloon pump (IABP), surgeons tend to use the assist device. If echocardiography fails to reveal a surgically correctable cause for cardiogenic shock, most surgeons use homodynamic data to consider the need for mechanical assistance. These criteria include a cardiac index less than 2.2 L/min/m^2^, systolic pressure lower than 90 mmHg, central venous pressure (CVP) higher than 20 mmHg, and concomitant use of a high dose of at least two inotropic agents.^3^^-^^5^ This situation may be clinically associated with arrhythmia, pulmonary edema, and oliguria. In such circumstances, the use of an IABP may be considered as the first step in the post-cardiotomy shock setting. Without mechanical support, mortality is greater than 50%. In this setting, some believe that early implantation of an assist device capable of supporting high flow and allowing the heart to rest may improve the outcome and allow for the recovery of stunned myocardium.^6^^, ^^7^

Since their invention in 1985, there have been more than 1000 cases of implanted ventricular assist devices for post-cardiotomy shock. Most of the recipients had ischemic heart disease; however, there were also rare cases of valvular heart disease with post-cardiotomy shock treated with bio-pump assistance. The average duration of support for these devices was approximately 3 days, 45% of the reported patients were weaned from circulatory assistance, and 25% of all the patients survived to discharge. These numbers remain consistent with previously reported statistics.^8^^, ^^9^ The results of the Biomedicus centrifugal pump, employed in post-myocardial infarction cardiogenic shock, remain limited. In the literature, of the 96 patients reported by 10 referral hospitals, 26% were weaned from support and only 11.5% survived to discharge.^10^^-^^12^

In our hospital, we utilized the bio-pump for first time in post-cardiotomy right heart failure resulting from mitral valve surgery. These patients did not respond to maximum inotropic agent and IABP. The mean ejection fraction of our cases was 42% ± 6%, and the mean pulmonary artery pressure was 50 ± 8 mmHg. Additionally, the mean end systolic volume in 2 cases was 76 ± 6 ml. For these patients, with continued cardiogenic shock despite maximum inotropic drug and IABP use, the bio-pump assist device was advocated. The cause of cardiogenic shock was post-cardiotomy severe RV failure. RV failure occurred after mitral valve replacement (one case of pure mitral regurgitation and three cases of mitral regurgitation and mitral stenosis). The mean pressure with the IABP and assist device was 63 ± 6 mmHg versus 20 ± 30 mmHg with the IABP off. During the first postoperative hour, the bio-pump flow ranged from 3.6 to 2.5 L/ml. The duration of the IABP use ranged from 5 to 10 days, and the duration of the bio-pump use ranged from 24 to 36 hours. All the 4 patients were successfully weaned from the assist device; nevertheless, one of these patients died after successful weaning from the RV assist device (RVAD) from cerebrovascular accident. One patient developed hemiparesis, which completely cleared after 3 months. All the survived patients developed pulmonary edema and adult respiratory distress syndrome, which were managed via tracheostomy and respiratory physiotherapy. One patient was readmitted due to sepsis and a splenic abscess in the second postoperative month. One patient was treated for lobar pneumonia and another for purulent tracheitis. 

A combined usage of the IABP and the bio-pump appears to be a better therapy than either one individually.

***Technique***

The Biomedicus Pump used in the present study consists of valveless rotator cones that create a rotatory motion in the incoming blood by viscous drag and constrained vortex principles. In this manner, the cones generate pressure and flow. These elements are contained in a polycarbonate cone-shaped container with an inlet and outlet. These pumps are afterload-dependent unlike the roller type pump. They can be used to bypass both the right and left ventricle and are easy to operate. They are driven by a magnetic impeller that has no direct contact with the blood within. A separate console allows the operator to control the revolutions and set alarms. Conventional bypass cannulae are used together with bypass tubing to connect the ventricle to the pump outside the body. The right atrial cannula (usually 32F or 34F venous cannula) can be placed through the right atrial appendage. Typically, purse string sutures with Teflon pledgets are placed in the base of the appendage, and passed through rubber tourniquets. The right atrial pressure is raised by restricting the venous return, the atrium is opened, and the cannula is inserted. After the right atrial cannula is secured, it is connected to the inflow line of the bio-pump. All air must be excluded from the connection and tubing at this time.

A pulmonary artery cannula (22F-angle aortic arch cannula) is inserted into the pulmonary artery distal to the pulmonic valve and secured with purse string sutures. The cannulae are brought out through the inferior angle of the incision. The skin is approximated, whereas the sternum is left open. The perfusion lines are securely sutured to the skin and covered with several towels and a large adhesive plastic drape. Weaning is usually not attempted during the first 24 hours of assist support. When the patient’s hemodynamic is stable and cardiac improvement is suspected, a weaning assessment can be completed within a few minutes or less. The following parameters must be assessed and recorded by the perfusionist: the pump flow must never be decreased to less than 1 L/min. At 1 L/min, the activated clotting time must be greater than 200 seconds, and the duration of time at this flow must not exceed 2 minutes. If the pump flow is to be kept at 1 L/min for longer than 2 minutes, the activated clotting time must be extended to 200 seconds.

***Anticoagulation Regimen and Coagulopathy***

After cardiopulmonary bypass (CPB), heparin should be reversed with protamine. After being returned to the Intensive Care Unit (ICU), the patient is not anticoagulated until clotting measures have normalized, after which the administration of heparin is commenced to maintain the activated clotting time above 150 seconds. The perfusionist on duty is responsible for performing activated clotting time monitoring and determining necessary heparin doses.^6^^-^^11^

***Case # One***

The patient was a 51-year-old woman who was referred from rural regions of Kurdistan with severe mitral stenosis associated with a left arterial clot and severe pulmonary hypertension. 

She underwent mitral valve replacement and removal of the clot. After having been weaned from CPB, the patient developed RV failure. CPB was, therefore, resumed and followed by a high dose of inotropic drugs and the IABP, which failed to yield complete response, and repeated ventricular tachycardia and fibrillation complicated the condition. Consequently, RV support through the insertion of a centrifugal pump was considered, and the patient was successfully weaned from CPB on the second postoperative day. The RV support was discontinued on the third postoperative day, and the IABP was removed without marked homodynamic changes on the seventh postoperative day. 

***Case # Two***

The patient was a 51-year-old woman who was referred to our center for the management of mitral stenosis associated with a large left atrial clot. 

She underwent the operation via CPB and moderate hypothermia. Antegrade cold cardioplegia was instituted to arrest and protect the heart and systemic hypothermia to 28 ^°^C. The left atrium was approached via the conventional method. The mitral valve was heavily calcified, and the subvalvular structures were fused. The anterior mitral leaflet was resected, while the posterior leaflet and its subvalvular complex were left intact. A 29-mm Carbomedix mitral prosthesis was implanted using separate pledged sutures. The patient was successfully weaned with a high dose of adrenalin and Dobutamine 60 minutes after CPB institution. 

The patient developed a low cardiac output in the immediate postoperative period. As the patient’s condition worsened progressively, the IABP was inserted. The homodynamic of the patient recovered transiently for a short time, but it was worsened subsequently by fibrillation and ventricular tachycardia, both of which were managed by cardioversion and intravenous Amiodarone administration. The systolic blood pressure was 60-70 mmHg. Episodes of paroxysmal ventricular tachycardia and occasional ventricular fibrillation reoccurred and became more frequent during the ensuing hours. High-dose inotrope support, including adrenalin (100 µ/kg/min), Dobutamine (40 µ/kg), and Milrinone (0.5 µ/kg), was commenced.

Informed consent was obtained to use a Biomedicus bio-pump as an RVAD so as to support the patient’s poor homodynamic condition. Four hours after the insertion of the RVAD, the patient’s homodynamic recovered. The RVAD’s output dung this time ranged from 2.3 to 3.2 L/min, and the systolic blood pressure ranged from 70 to 100 mmHg. No episode of paroxysmal ventricular tachycardia and fibrillation was observed. Right atrial pressure before the insertion of the RVAD was 35 mmHg, and it dropped to 15 mmHg on the second postoperative day. Postoperative creatinine phosphokinase myocardial band (CPK-MB) value was normal, which ruled out RV infarction due to poor protection or myocardial infarction. Hepatic enzymes and direct and indirect bilirubin values were elevated, and urine output was maintained with a high dose of Lasix. On the third postoperative day, the pump flow was gradually decreased to 500/ml/minute for 6 hours without major hemodynamic changes. Improved left ventricular contraction and remarkably improved RV contractility were detected in transesophageal echocardiography (TEE). Thereafter, the device was minimized to slow flow, and the patient was subsequently weaned from the device after 72 hours. The sternum was left open after subsequent device removal with skin closure until the fifth postoperative day. All the inotropic drugs were progressively reduced and discontinued. During this time period, hemolysis, jaundice and bleeding occurred; they were all treated with large amounts of packed red cells and other blood products. The IABP was discontinued on the seventh postoperative day.

The patient developed adult respiratory distress syndrome, which was managed via tracheostomy and intense respiratory care. She was weaned from the ventilator on the thirteenth postoperative day, and her renal and hepatic function as well as neurologic status had completely recovered by the sixteenth postoperative day. 

***Case # Three***

The patient was a 37-year-old woman with a past medical history of mitral valve replacement. She was referred to our hospital for the management of prosthetic mitral valve malfunction. 

The operation was conducted via CPB with moderate hypothermia and antegrade and retrograde cardioplegia injections. The left atrium was opened via the conventional method. The prosthetic mitral valve had an old pannus tissue with recent thrombosis in its ring. The prosthetic valve was cleaned, and the left atrium was irrigated with copious amounts of normal saline. The patient was successfully weaned from CPB with high-dose inotropic drugs, but she developed a low cardiac output in the immediate postoperative period. As the patient’s condition worsened and did not respond to balloon pump and high-dose inotropic drug support, we decided to use a bio-pump as an RVAD in order to support the patient’s poor hemodynamic condition. After the application of a Biomedicus centrifugal pump as an RVAD, the patient was transferred to the ICU with the IABP, bio-pump, and high-dose inotropic drug support. 

Three hours after the insertion of the bio-pump, the RV tachycardia was abolished. During this time period, the bio-pump’s output was reduced from 4 L/min to 3 L/min. The mean and systolic blood pressures were 70 and 90 mmHg, respectively. The patient’s high CVP (37 mmHg) in the first hour following the insertion of the bio-pump reduced to 15 mmHg on the second day. 

After 30 hours of bio-pump use, we reduced the bio-pump’s flow to a minimum of 500 ml/min and reduced the patient’s adrenaline and Dobutamine requirement to the minimum dose. During this time period, cardiac enzymes, including CPK-MB, were normal, but the patient developed severe jaundice and bleeding, which were treated with 30 unites of packed red cells and blood products. Postoperative acute respiratory distress syndrome was managed via tracheostomy and respiratory care. On the seventh postoperative day, the patient was completely weaned from ventilation.

***Case # Four***

The patient was a 33-year-old woman with a past medical history of open mitral commissurotomy. She was referred to our center for the management of combined severe mitral regurgitation and stenosis. 

The operation was conducted via CPB and hypothermia (28 ^°^C), and cardiac protection was achieved with an antegrade cardioplegia infusion. The left atrium was approached via the *transseptal* method. Following the resection of the heavily calcified and tethered mitral valve apparatus, the mitral valve was replaced with a Carbomedics valve (29 mm) using continuous sutures of 2/0 viline and the tricuspid valve was repaired via the Devega method. The patient was successfully weaned from CPB with high-dose inotropic drug and IABP support. During the following 6 hours, the RV function did not improve and the inotropic support could not be reduced. 

At this stage, the patient developed signs of end-organ dysfunction, elevated transaminase levels, and a decreased urine output. We, therefore, implanted a Biomedicus bio-pump as an RVAD. Eight hours after the insertion of the RVAD, the patient’s hemodynamic recovered, the RVAD’s flow (4 lit/min) dropped to 3 lit/min, and the right atrial pressure reduced from 40 mmHg to 15 mmHg. Cardiac enzymes were normal, and the patient was weaned from the respirator on the seventh post-RVAD insertion day. 

The patient’s renal failure necessitated continuous peritoneal dialysis for 2 days. Antimicrobial therapy with intravenous Ceftazidime and Gentamicin was also commenced. In the following days, the signs of end-organ dysfunction improved, as was indicated by an increase in the urine output, resolution of jaundice, and reduction in hepatic enzymes levels. Two days after the patient had been weaned from the mechanical respirator, the IABP was removed. Unfortunately, however, the patient died due to fatal intracerebral hemorrhage. The hemodynamic values of the 4 patients are depicted in Table 1.

**Table 1:** Hemodynamic values in four patients weaned from cardiopulmonary bypass with Biomedicus bio-pump^*^

Value	Before BBI	During BBI	After BBI
SAP (mmHg)	63.8±0.83	63.3±1.1	77.3±1.5
PAP (mmHg)	45.1±1.6	47.6±1.2	21.8±1.5
RAP (mmHg)	30.7±1.2	33.5±2.4	16.5±1.2

SAP, Systolic arterial pressure; PAP, Pulmonary arterial pressure (systolic); BBI, Biomedicus bio-pump insertion; RAP, Right atrial pressure

* Data are presented as mean±SD

## Discussion

The optimal management for patients with profound right cardiac failure and malignant ventricular arrhythmias after valve replacement associated with severe pulmonary hypertension has yet to be clearly delineated. The available options include the biventricular assist device, the left ventricular assist device, and the RVAD. Since its invention in 1985, the ventricular assist device has been implanted in more than 1000 patients for the management of post-cardiotomy cardiogenic shock. Most of the recipients had ischemic heart disease, and a few cases received the bio-pump as an RVAD for the treatment of post-valvular cardiotomy shock. The average duration of support for these devices is approximately 3 days. Forty-five per cent of the reported patients were weaned from circulatory assistance, and 25% of all the patients survived to discharge. These numbers remain consistent with previously reported statistics. 

The results of the bio-pump as an RVAD for the treatment of post-myocardial infarction cardiogenic shock remain limited. In the literature, of the 96 patients reported by 10 referral hospitals, 26% were weaned from support and only 11.5% survived to discharge. In our hospital, we used the bio-pump for the first time for the treatment of post-cardiotomy right heart failure in patients undergoing valvular surgery. None of our patients responded to maximum inotropic agents and IABP support. The mean ejection fraction in our cases was 35% ± 5%, and the mean pulmonary artery pressure was 60 ± 8 mmHg. The end systolic volume in 2 cases was 76 ml. We used the bio-pump in 4 patients (3 women and one man, age ranging from 33 to 60 years old) with refractory and cardiogenic shock not responsive to inotropic drug use and the IABP insertion. The cause of cardiogenic shock was myocardial infarction in one case (severe mitral valve calcification) and stunning or inadequate myocardial protection in the other 3 patients (one case of pure mitral regurgitation and 2 cases of combined mitral regurgitation and mitral stenosis). The mean arterial pressure with the IABP and the assist device was 63 ± 6 mmHg versus 20-30 mmHg with the IABP off. In the first hours following the insertion of the bio-pump, the flow ranged from 3.6 to 2.5 lit/ml. The duration of the IABP use ranged from 5 to 10 days.

The artificial heart is not available at our institution; as a result, we employed the Biomedicus bio-pump as an RVAD in our 4 patients for the following reasons:^12^^-^^14^ 1. severe pulmonary hypertension (110 mmHg); 2. high right atrial pressure (35 mmHg); 3. low left atrial pressure; 4. poor homodynamic condition (systemic hypotension); 5. RV distension; 6. good left ventricular function or vigorous left ventricular contraction; and 7. refractory arrhythmia.

We also drew upon the following echocardiographic criteria for the definition of the RV dysfunction after open heart surgery: tricuspid valve regurgitation; RV end diastolic diameter (RVEDD) > 40 mm; RV ejection fraction (RVEF) < 30%; and right atrial dimension > 50 mm.

Previous experience and published information indicate that the RVAD is useful in RV infarction following open heart surgery.^6^^-^^14^ Be that as it may, there is no published information about ventricular arrhythmia and right heart failure secondary to severe pulmonary hypertension treated with the RVAD. We assumed that right heart failure and ventricular arrhythmia were an acute event that would be resolved by reducing pulmonary hypertension as indicated by postoperative echocardiography. Our patients had ventricular arrhythmia that at times repeated, and they experienced numerous cardioversions and defibrillation with no adverse sequel. The placement of the RVAD in a second operation was considered because we believed the arrhythmias and poor homodynamic condition would resolve with time in these patients. Circulatory support using only a centrifugal pump as an RVAD and the IABP was feasible despite refractory and recurrent ventricular arrhythmias and low output syndrome. Although RV failure due to severe pulmonary hypertension is a serous intraoperative complication, favorable results were obtained by combined therapy with the IABP and the RV support using a centrifugal pump in our patients.^15^^-^^17^

## Conclusion

Isolated RV failure is diagnosed on the basis of low left atrial and systemic arterial pressures and elevated right atrial pressure in addition to vigorous left ventricular contractions, right ventricular distention, and severe RV dysfunction as detected by transesophageal echocardiography. Standard therapy for severe right heart failure consists of inotropic drug support, volume unloading, and application of pulmonary vasodilators (prostaglandin and nitric oxide). When pharmacological agents are unable to improve the RV contraction, surgeons must rely on mechanical means to restore the blood flow to the pulmonary circulation and left ventricle.

## Notes

***This paper should be cited as:*** Moulodi A. R, Sheibat Zadeh G. R, Sabzi F. The Use of Right Ventricular Support with a Centrifugal Pump in Post Valve Surgery Right Ventricular Failure: A Case series. J Teh Univ Heart Ctr 2014;9(1):*38-42*
